# Impact of Antibiotic Prophylaxis on Infection Rate after Endoscopic Ultrasound Through-the-Needle Biopsy of Pancreatic Cysts: A Propensity Score-Matched Study

**DOI:** 10.3390/diagnostics12010211

**Published:** 2022-01-16

**Authors:** Antonio Facciorusso, Martha Arevalo-Mora, Maria Cristina Conti Bellocchi, Laura Bernardoni, Daryl Ramai, Paraskevas Gkolfakis, Domenico Loizzi, Nicola Muscatiello, Antonio Ambrosi, Nicola Tartaglia, Carlos Robles-Medranda, Elisa Stasi, Andrew Ofosu, Stefano Francesco Crinò

**Affiliations:** 1 Gastroenterology Unit, Department of Medical and Surgical Sciences, University of Foggia, 71122 Foggia, Italy; antonio.facciorusso@virgilio.it (A.F.); nmuscatiello@libero.it (N.M.); 2 Gastroenterology and Digestive Endoscopy Unit, Department of Medicine, The Pancreas Institute, University Hospital of Verona, 37134 Verona, Italy; mcristina.contibellocchi@gmail.com (M.C.C.B.); laura.bernardoni@aovr.veneto.it (L.B.); 3 Instituto Ecuatoriano de Enfermedades Digestivas, Guayaquil 090505, Ecuador; martha_am@hotmail.com (M.A.-M.); carlosoakm@yahoo.es (C.R.-M.); 4 Division of Gastroenterology and Hepatology, University of Utah Health, Salt Lake City, UT 84112, USA; Daryl.Ramai@hsc.utah.edu; 5 Department of Gastroenterology, Hepatopancreatology, and Digestive Oncology, CUB Erasme Hospital, Université Libre de Bruxelles (ULB), 1050 Brussels, Belgium; pgolfakis@gmail.com; 6 Thoracic Surgery Unit, Department of Medical and Surgical Sciences, University of Foggia, 71122 Foggia, Italy; domenico.loizzi@unifg.it; 7 General Surgery Unit, Department of Medical and Surgical Sciences, University of Foggia, 71122 Foggia, Italy; antonio.ambrosi@unifg.it (A.A.); nicola.tartaglia@unifg.it (N.T.); 8 Gastroenterology and Digestive Endoscopy, ‘Vito Fazzi’ Hospital, 73100 Lecce, Italy; elisastasi2@gmail.com; 9 Division of Digestive Diseases, University of Cincinnati, Cincinnati, OH 45221, USA; andyofosu@gmail.com

**Keywords:** endoscopic ultrasound, through-the-needle biopsy, pancreas, antibiotics

## Abstract

Background: Despite weak evidence, antibiotic prophylaxis prior to endoscopic ultrasound-guided through-the-needle biopsy (EUS-TTNB) of pancreatic cystic lesions (PCLs) is routinely used in clinical practice. We aim to compare a group of patients treated with antibiotics before EUS-TTNB of PCLs and a group who did not undergo antimicrobial prophylaxis. Methods: Out of 236 patients with pancreatic cystic lesions referred to two high-volume centers between 2016 and 2021, after propensity score matching, two groups were compared: 98 subjects who underwent EUS-TTNB under antibiotic prophylaxis and 49 subjects without prophylaxis. Results: There was no difference in terms of baseline parameters between groups. Final diagnosis was serous cystadenoma in 36.7% of patients in the group not treated with prophylaxis and in 37.7% of patients in the control group, whereas IPMN and mucinous cystadenoma were diagnosed in 3 (6.1%) and 16 (32.6%) versus 6 (6.1%) and 32 (32.6%) patients in the two groups, respectively (*p* = 0.23). Overall, the adverse event rate was 6.1% in the group not treated with antibiotic prophylaxis and 5.1% in the control group (*p* = 0.49). Only a single infectious adverse event occurred in each group (*p* = 0.48). The diagnostic yields were 89.7% and 90.8% in the two groups (*p* = 0.7), and the diagnostic accuracy rate was 81.6% in both groups (*p* = 1.0). Conclusions: Prophylactic antibiotics do not seem to influence the risk of infection, and their routine use should be discouraged.

## 1. Introduction

The incidence of pancreatic cystic lesions (PCLs) is increasing due to improvements in diagnostic imaging, with a reported prevalence ranging from 2% to 16% mainly depending on patient age [1].

Given the malignant potential of certain subtypes of PCLs, such as mucinous cystic neoplasms (MCN) and intraductal papillary mucinous neoplasms (IPMN), a correct diagnostic definition of these lesions is mandatory, and morphology alone is frequently not sufficient in discriminating neoplastic from non-neoplastic PCLs [2,3,4]. To this end, EUS-guided tissue sampling of PCLs is commonly used to improve diagnostic accuracy.

Unfortunately, standard EUS-guided fine-needle aspiration (FNA) has not proven to be reliable in accurately discriminating the type of lesion and the risk of malignancy, with a reported sensitivity as low as 54% for differentiating mucinous from non-mucinous cysts [5,6]. 

Despite encouraging results observed with molecular analysis of cystic fluid [7] and with novel devices such as confocal laser endomicroscopy [8], high costs and limited experience make these techniques a niche diagnostic tool that is not available worldwide.

Recently, a through-the-needle microforceps biopsy (TTNB) device (Moray Microforceps^®^, US Endoscopy, Mentor, OH, USA) that can be passed through a standard 19-gauge EUS-FNA needle was developed for histologic sampling of PCLs [9]. Despite the highly favorable diagnostic performance, as demonstrated in recent meta-analyses reporting adequacy and accuracy rates of 85.3% and 78.8%, respectively [10,11], safety concerns have limited its use in clinical practice. Particularly, the safety concerns raised in a recent European study prompted the use of prophylactic measures such as the use of rectal indomethacin and antibiotic prophylaxis [12].

However, the real effectiveness of these measures is still unclear; therefore, the use of antibiotic prophylaxis (usually with fluoroquinolones or beta-lactams) is based only on long-standing clinical practice and very limited evidence. 

Several studies [13,14] and a recent meta-analysis [15] demonstrated that antibiotic prophylaxis is not needed to prevent infections after EUS-FNA of PCLs; however, the results of these studies should not be considered applicable to EUS-TTNB. This is due to differences in technical features such as the use of larger needles, the longer procedural time, and the higher risk of intra-cystic bleeding, with incomplete cysts emptying that may represent indications for antibiotic prophylaxis with this device.

Since the use of antimicrobial agents increases the cost of the procedure as well as the risk of drug resistance, determining the real benefit of this practice is of paramount importance in drawing definitive conclusions on this topic. 

The aim of our study was to compare patients treated with antibiotics before EUS-TTNB of PCLs and a group who did not undergo antimicrobial prophylaxis through a propensity score matching model.

## 2. Materials and Methods

### 2.1. Patients

Data of consecutive patients who underwent EUS-TTNB at four high-volume centers from January 2016 to June 2021 were reviewed. Exclusion criteria included age <18 years, extra-pancreatic lesions, and less than one month of follow-up. 

The main indications for EUS-TTNB were unilocular/oligocystic lesion without communication with the main pancreatic duct, cyst size greater than 3 cm, main pancreatic duct diameter of 5 mm or greater, increased dimension/changes in morphology during follow-up, thickened walls/mural nodules, and increased CA19.9. Contraindications to EUS-TTNB were lactating and pregnant women, patients with uncorrected coagulopathy (international normalized ratio > 1.5 or platelet count <50 × 109/L), and history of recent pancreatitis (within last 3 months).

All procedures were performed by experienced board-certified endoscopists, with at least 5 years of experience with EUS-guided tissue sampling of PCLs. The study population initially included two groups of patients: 187 who underwent EUS-TTNB under antibiotic prophylaxis with 200 mg of ciprofloxacin (Ciproxin^®^, Bayer, Germany) administered intravenously over 30 min prior to the procedure followed by 3–5 days of oral ciprofloxacin before November 2020 and 49 treated with no antimicrobial agents from December 2020 onward. 

Institutional Review Board approbation for this retrospective report was obtained (N. 3373CESC, 12 July 2021). 

### 2.2. Procedures

Endoscopic procedures were performed with patients under propofol sedation. Following initial EUS evaluation, the lesion was punctured with a 19G EUS-FNA needle, with the microbiopsy forceps preloaded or subsequently introduced through the needle after removal of the stylet. Targeted biopsies were performed in mural nodules or wall thickening (if present) or, alternatively, in the cyst wall. 

Prophylaxis of pancreatitis with rectal indomethacin/diclofenac was used in all patients. Similarly, the number of microforcep passes was not standardized and depended on the endoscopists’ discretion. In the absence of technical issues, the TTNB procedure was performed with a single needle pass and an attempt to complete aspiration of the cyst fluid was performed in all cases. Patients were observed for a few hours after the procedure and discharged upon uneventful recovery. 

### 2.3. Follow-Up and Outcomes

All clinical and safety outcomes were assessed by clinicians blinded to the prophylactic strategy adopted. Adverse event (AE) rates were evaluated during the procedure, before discharge, and at 1 and 7 days by means of telephone calls. All AEs were classified according to ASGE lexicon [16]. A severe adverse event was defined as one that required hospitalization, was life-threatening, or resulted in death or disability.

Primary variables included infection of the pancreatic cyst, defined as fever (>38 °C for >24 h), after EUS-TTNB. Secondary variables included other complications of the procedure or related to the use of prophylaxis (i.e., allergic reactions). Timing of AE occurrence was classified as intraprocedure, ≤14 days, or >14 days [16].

Secondary endpoints were diagnostic yield, defined as the percentage of lesions sampled for which a tissue diagnosis was obtained, and diagnostic accuracy was defined as the percentage of lesions that correspond to the final diagnosis at surgical histopathology. The gold standard for diagnosis was considered surgical histology whenever available. In non-resected patients, the final diagnosis was established on a combination of cross-sectional imaging/EUS findings and TTNB histology/cyst fluid cytology as previously defined [17].

### 2.4. Statistical Analysis

Categorical variables were reported as the number of cases and percentage, and differences between groups were compared using the Chi-square and McNemar analysis before and after matching, respectively.

Continuous variables were expressed as median and interquartile range (IQR), and differences between groups were explored by the Mann–Whitney and Wilkoxon-rank tests before and after matching, respectively. All analyses were two-tailed, and the threshold of significance was assessed at <0.05. 

To overcome biases owing to the different distributions of covariates among patients who were submitted to EUS-TTNB with or without antibiotic prophylaxis, a 1-to-2 match was created using propensity score analysis. The propensity score represents the probability of each individual patient being assigned to a particular condition in a study given a set of known covariates [18].

A multivariate logistic regression was built to predict the probability of each individual patient being submitted to the two groups on the basis of covariates that are known to be able to affect postoperative outcomes, namely age, gender, Charlson comorbidity index, body mass index (BMI), symptoms, indications to the procedure, lesion location and size, number of needle and microforceps passes, complete aspiration of the cyst, and final diagnosis. The predictive values were then used to obtain a 1-to-2 match by using nearest neighbor matching within a specified caliper distance. Nearest neighbor matching within a specified caliper distance matches an untreated subject whose propensity score is closest to that of the treated subject (“nearest neighbor matching” approach) with the further restriction that the absolute difference in the propensity scores of matched subjects must be below some pre-specified threshold (the caliper distance) [19,20]. Thus, patients for whom the propensity score could not be matched due to greater caliper distance were excluded from further analysis. As suggested by Austin, a caliper of width equal to 0.2 of the standard deviation of the logit of the propensity score was used, as this value has been found to minimize the mean squared error of the estimated treatment effect [19]. 

Based on a previous meta-analysis [10], an infectious adverse event rate of 1% after EUS-TTNB was expected. To demonstrate the non-inferiority of no antibiotic prophylaxis, based on a statistical power of 80%, a significant level of 5%, and a non-inferiority limit of 5%, a sample size with 49 patients in the non-prophylaxis group and 98 in the antibiotic prophylaxis group was needed.

The statistical analysis was performed using the MatchIt package in R Statistical Software 3.0.2 (Foundation for Statistical Computing, Vienna, Austria).

## 3. Results

### 3.1. Patients

**Figure 1:**
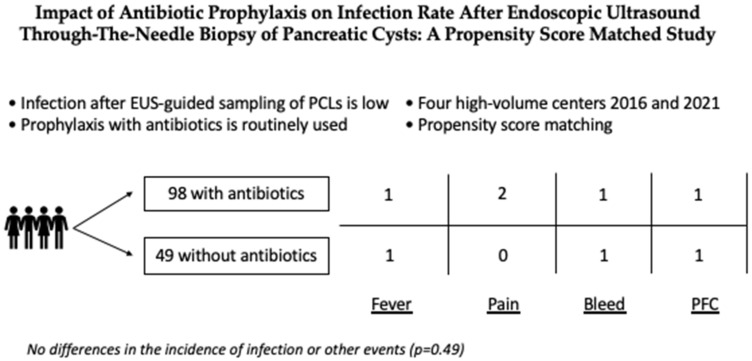
Pictorial description of the design of the study. PFC, pancreatic fluid collection.

Out of 236 patients with pancreatic cystic lesions sampled with EUS-TTNB, 187 underwent EUS-TTNB with antibiotic prophylaxis and 49 without prophylaxis. After propensity score matching, two groups were compared: 98 subjects who underwent EUS-TTNB under antibiotic prophylaxis and 49 sampled without prophylaxis. A pictorial description of the study design is reported in Figure 1. 

**Figure 2:**
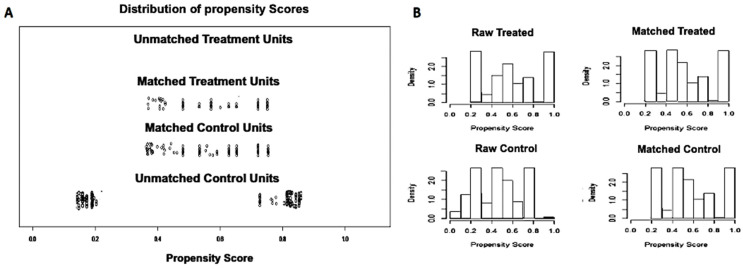
Propensity score matching. Out of 236 patients with pancreatic cystic lesions, after propensity score matching two groups were compared: 98 subjects who underwent through-the-needle biopsy under antibiotic prophylaxis and 49 sampled without prophylaxis. (**A**). Propensity score matching jitter plot. (**B**). Propensity score matching histogram.

Baseline characteristics of the two matched sample groups are reported in Table 1, and details of the propensity score matching are depicted in Figure 2.

Median age was 62 (46–75) years in the group not treated with antibiotic prophylaxis versus 63 (48–76) in the control group (*p* = 0.79), and 26.5% of patients were male in both groups (*p* = 1.0). Median Charlson comorbidity index and BMI were 4 (2–5) and 24 (21–30) in the group not treated with antibiotic prophylaxis and 4 (2–5) and 23 (20–28) in the control group, respectively (*p* = 0.4 and 0.51). The majority of patients were asymptomatic in both study groups (*p* = 0.37), whereas the main indication to EUS-TTNB was the presence of a PCL >3 cm (61.2% and 59.1% in the two groups, respectively; *p* = 0.28). Most of the lesions were in the pancreatic head, with a median size of 33 (27–41) mm in the group of patients not treated with antibiotic prophylaxis versus 35 (29–40) mm in the control group (*p* = 0.76). Unilocular cysts were observed in 34 (69.3%) and 68 (69.3%) patients in the two groups, respectively (*p* = 1.0), and the majority of lesions (69.3% in both groups) were sampled from the stomach (*p* = 0.98).

Median number of needle passes and microforceps passes was 1 (1–2) and 2 (1–3) in both groups (*p* = 0.54).

The cyst was aspirated completely in 81.6% and 79.5% of patients in the two groups (*p* = 0.87). Prophylaxis with rectal indomethacin/diclofenac was used in 16.3% of patients, without a difference between groups (*p* = 0.98).

Median follow-up lengths were 17 (10–25) months in the group without antibiotic prophylaxis and 18 (12–26) in the control group (*p* = 0.74).

### 3.2. Adverse Events

**Figure 3:**
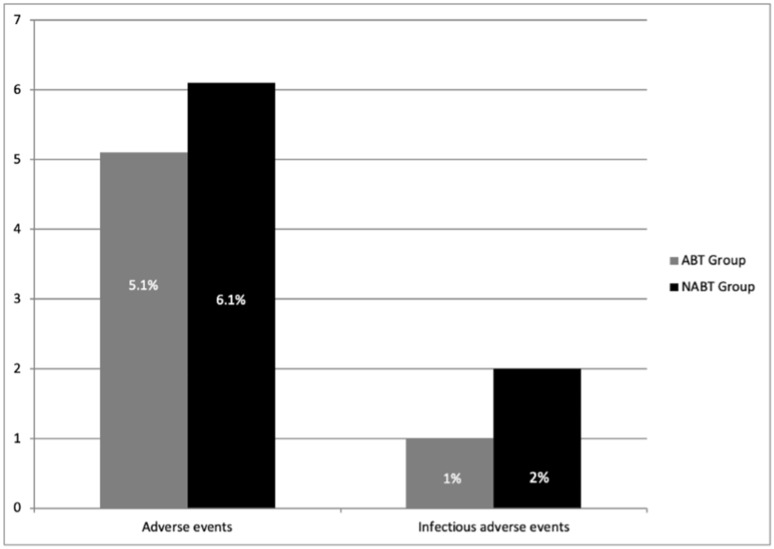
Comparison of overall and infectious adverse events between the two study groups.

A detailed list of the study outcomes is reported in Table 2. Adverse event rate was 3/49 (6.1%) in the group without antibiotic prophylaxis and 5/98 (5.1%) in the control group (*p* = 0.49; Figure 3).

The adverse events observed in the group not treated with antibiotic prophylaxis were a peri-pancreatic fluid collection that occurred 20 days after EUS-TTNB, a case of fever and mild bleeding. The collection was treated with EUS-guided drainage by means of a 10 × 10 mm metal stent (Hot-Axios^®^, Boston Scientific, Marlborough, MA, USA), which was removed after 15 days upon full recovery of the patient. On the other hand, the adverse events observed in the control group were two cases of mild abdominal pain occurring the day after the procedure and were managed conservatively, one case of mild bleeding, and one case of fever and peri-pancreatic fluid collection that occurred 16 days after the procedure and was drained with a 10 × 10 mm metal stent.

As a consequence, no serious adverse events were observed in any of the two study groups and no cases of antibiotic-related allergic events were registered (Table 2).

### 3.3. Secondary Outcomes

The diagnostic yields were 89.7% and 90.8% in the two groups, respectively (*p* = 0.7), and the diagnostic accuracy rate was 40/49 (81.6%) in the group not treated with antibiotic prophylaxis versus 80/98 (81.6%) in the control group (*p* = 1.0).

Final diagnosis was serous cystadenoma in 18 patients (36.7%) in the group not treated with prophylaxis and in 37 patients (37.7%) in the control group, whereas IPMN and mucinous cystadenoma were diagnosed in 3 (6.1%) and 16 (32.6%) versus 6 (6.1%) and 32 (32.6%) patients in the two groups, respectively (*p* = 0.23).

Surgical confirmation of final diagnosis was obtained in 9 patients (18.3%) in the first group versus 17 patients (17.3%) in the control group (*p* = 0.56).

## 4. Discussion

Although the rate of infectious adverse events after EUS-guided sampling of PCLs is low [21], prophylaxis with fluoroquinolones or beta-lactam antibiotics is routinely used in most centers and the current guidelines recommend this practice, albeit supported only by very limited experience [5].

This strategy has been recently questioned in the setting of EUS-FNA of PCLs based on several retrospective studies [14,22] and a large RCT [13] who failed to find a significant difference between the two approaches in terms of infectious adverse events, as confirmed also in a recent meta-analysis [15].

However, the results of these studies should not be considered applicable to EUS-TTNB where the use of larger needles, longer procedural time, higher risk of intracystic bleeding, or the higher risk of incomplete cyst emptying might still represent indications to antibiotic prophylaxis.

To the best of our knowledge, our study represents the first series comparing antibiotic prophylaxis versus no prophylaxis in patients undergoing EUS-TTNB of PCLs.

To overcome the potential biases related to the retrospective nature of the study and to account for confounding variables, we performed a propensity score matching analysis on the basis of covariates that are known to be able to affect post-procedural outcomes. Thus, two perfectly balanced treatment groups were obtained (Table 1).

Only one case of infectious events was registered in each group, and the only moderate events detected were one case of peri-pancreatic fluid collection per group. Therefore, no difference was observed between groups, thus supporting evidence against the routine use of antibiotic prophylaxis. Of note, even the two moderate adverse events were resolved successfully without permanent consequences for the patients.

The routine use of antibiotics presents several negative implications, namely it increases the cost of the procedure and the risk of drug resistance, it might be associated with potentially serious allergic reactions and secondary infections, although none of them were registered in our series probably due to the limited sample size. Moreover, complex regimens involving parenteral antibiotics hours in advance or oral courses after the procedure increases the complexity of the procedure and may result in non-adherence. Furthermore, while ciprofloxacin is well tolerated, the cost for a 2-week course ranges from USD 17 (generic) to USD 214 (brand).

Therefore, as already demonstrated with EUS-FNA, EUS-TTNB should also be considered a technique burdened with very low risk of infectious adverse events. On the other hand, serious adverse events other than infections were reported in previous studies [12], thus limiting the widespread in the use of this device. Hence, proper patient selection should be mandatory in this setting, limiting the use of EUS-TTNB in higher-risk patients, particularly IPMN patients [23,24].

Of note, complete aspiration of the PCL was not possible in about 20% of patients in both groups. It could be postulated that incomplete aspiration of the cyst could increase the risk of adverse events due to the potential higher risk of infection and to the correlation between incomplete aspiration and intracystic bleeding. Moreover, some inflammatory events with edema/microhemorrhage close to the cystic wall due to the TTNB trauma are conceivable. It could be postulated that complete aspiration of the cyst could minimize the pressure of edema/microhemorrhage against the pancreatic parenchyma nearby the cyst, thus reducing the risk of acute pancreatitis. However, the results of our study do not seem to support this potential increased risk although the limited sample size calls for a note of caution in the interpretation of these findings. Nevertheless, even in this potentially worse scenario of incomplete cyst aspiration, antibiotic prophylaxis did not seem to determine beneficial effects compared with no prophylaxis.

It should be noted that the overall rate of AEs was relatively low in our study, in line with a previous meta-analysis on the use of EUS-TTNB in patients with PCLs [10]. This finding confirms that EUS-TTNB represents a safe procedure if a proper selection of patients is adopted.

There are some limitations to our study. First, the retrospective nature of the study and the relatively limited sample size could have led to selection biases. However, a propensity score-matching analysis based on several covariates known to influence post-procedural outcomes was performed to obviate bias. Furthermore, a rigorous sample size calculation was performed considering a non-inferiority scenario, as already conducted in the setting of EUS-FNA of pancreatic cysts. Thus, the study groups were perfectly balanced without statistically different baseline parameters. Second, a cost-effectiveness analysis was beyond the scope of the present study and could not be addressed.

Despite such a limitation, we think that our analysis provides robust evidence supporting the absolute comparability between the two strategies (i.e., antibiotic prophylaxis versus no prophylaxis) in terms of infectious event prevention.

## 5. Conclusions

The incidence of infectious complications after EUS-TTNB of PCLs either with or without antibiotic prophylaxis appears very low, if proper patient selection is adopted. Consequently, since prophylactic antibiotics do not seem to substantially reduce this risk and routine use of prophylactic antibiotics is costly and not completely free of adverse events, our findings provide evidence against this practice. Broad prospective randomized trials are warranted to confirm the results of our analysis.

## Figures and Tables

**Table 1:** Baseline patient characteristics.

Variable	No Antibiotic Prophylaxis (*n* = 49)	Antibiotic Prophylaxis (*n* = 98)	*p* Value
Age (years)	62 (46–75)	63 (48–76)	0.79
Gender—Male	13 (26.5%)	26 (26.5%)	1.0
Charlson comorbidity index	4 (2–5)	4 (2–5)	0.4
Body mass index	24 (21–30)	23 (20–28)	0.51
Symptoms			
Asymptomatic	30 (61.2%)	64 (65.3%)	0.37
Upper abdominal pain	10 (20.4%)	19 (19.4%)	
Acute pancreatitis	3 (6.1%)	5 (5.1%)	
Jaundice	6 (12.3%)	10 (10.2%)	
Indications to the procedure			
Unilocular/oligocystic lesion without	3 (6.1%)	6 (6.1%)	
communication with MPD			
Size greater than 3 cm	30 (61.2%)	58 (59.1%)	0.28
Increased dimension	5 (10.2%)	6 (10.2%)	
Suspected mural nodules	4 (10.2%)	8 (10.2%)	
Thickened walls	7 (12.3%)	20 (14.4%)	
Lesion location			
Head	20 (40.8%)	39 (39.7%)	0.36
Body	13 (26.5%)	26 (26.5%)	
Tail	16 (32.7%)	33 (33.8%)	
Lesion size (mm)	33 (27–41)	35 (29–40)	0.76
Cyst type			
Unilocular	34 (69.3%)	68 (69.3%)	1.0
Oligocystic	15 (30.7%)	15 (30.7%)	
Cyst wall			
Thin	39 (79.5%)	80 (81.6%)	0.92
Thick	8 (16.3%)	14 (14.2%)	
Nodules	2 (4.2%)	4 (4.2%)	
Puncture site			
Stomach	34 (69.3%)	68 (69.3%)	0.98
Duodenal bulb	12 (24.4%)	24 (24.4%)	
Descending duodenum	3 (6.3%)	6 (12.6%)	
Number of needle passes	1 (1–2)	1 (1–2)	0.54
Number of microforceps passes	2 (1–3)	2 (1–3)	0.54
Complete aspiration of the cyst	40 (81.6%)	78 (79.5%)	0.87
Use of rectal indomethacin/diclofenac	8 (16.3%)	16 (16.3%)	0.98
Follow-up length (months)	17 (10–25)	18 (12–26)	0.74
Final diagnosis			
IPMN	3 (6.1%)	6 (6.1%)	0.23
Serous cystadenoma	18 (36.7%)	37 (37.7%)	
Mucinous cystadenoma	16 (32.6%)	32 (32.6%)	
Pseudocyst	2 (4%)	4 (4%)	
Neuroendocrine tumor	2 (4%)	4 (4%)	
PDAC	2 (4%)	5 (5.1%)	
Undefined	6 (12.6%)	10 (10.5%)	

Variables were reported as absolute numbers (percentage) or median (interquartile range) when appropriate.

**Table 2:** Outcomes.

Outcome	No Antibiotic Prophylaxis (49 pts)	Antibiotic Prophylaxis (98 pts)	*p* Value
Adverse event rate	3 (6.1%)	5 (5.1%)	0.49
Type of adverse event			
Abdominal pain	0 (0%)	2 (2%)	0.48
Peri-pancreatic fluid collection	1 (2%)	1 (1%)	
Fever	1 (2%)	1 (1%)	
Bleeding	1 (2%)	1 (1%)	
Severity adverse event			
Incident	0 (0%)	0 (0%)	0.61
Mild	2 (4%)	4 (4%)	
Moderate	1 (2%)	1 (1%)	
Severe	0 (0%)	0 (0%)	
Fatal	0 (0%)	0 (0%)	
Timing of adverse event occurrence			
Intraprocedure	1 (2%)	1 (1%)	0.43
<14 days	1 (2%)	4 (4%)	
>14 days	1 (2%)	0 (0%)	
Diagnostic yield	44 (89.7%)	89 (90.8%)	0.7
Diagnostic accuracy	40 (81.6%)	80 (81.6%)	1.0

## Data Availability

The datasets generated during and/or analyzed during the current study are available from the corresponding author upon reasonable request.
